# Pancreatic Cancer Screening in Patients with Type 2 Diabetes Mellitus: A Narrative Review

**DOI:** 10.3390/medicina62010067

**Published:** 2025-12-28

**Authors:** Mirela Dănilă, Ana-Maria Ghiuchici, Renata Bende, Iulia Rațiu, Felix Bende

**Affiliations:** 1 Department of Internal Medicine II, Division of Gastroenterology and Hepatology, “Victor Babeș” University of Medicine and Pharmacy, 300041 Timișoara, Romaniabende.felix@umft.ro (F.B.); 2 Center for Advanced Research in Gastroenterology and Hepatology, “Victor Babeș” University of Medicine and Pharmacy, 300041 Timișoara, Romania

**Keywords:** pancreatic ductal adenocarcinoma, type 2 diabetes mellitus, new-onset diabetes, targeted screening

## Abstract

Pancreatic ductal adenocarcinoma (PDAC) remains a high-burden disease worldwide with increasing incidence, poor prognosis, and high mortality. Complete surgical resection is the only potentially curative treatment; however, due to a lack of symptoms in the early stages, most patients have advanced disease when diagnosed. Type 2 diabetes mellitus (T2DM) is a significant health concern characterized by hyperglycemia, insulin resistance, and impairment in insulin secretion. T2DM is linked with PDAC, sharing a complex bidirectional relationship. Therefore, dual causality between the two diseases represents significant challenges in practice, distinguishing existing T2DM as a PDAC risk factor from newly onset, potentially pancreatic cancer-related diabetes (PCRD). Evidence showed that new-onset diabetes (NOD) may serve as a biomarker for early diagnosis of PDAC, and several risk prediction models were developed to identify high-risk patients for further intervention. Although early PDAC detection is important, widespread screening is not currently recommended for T2DM patients due to a lack of cost-effective, efficient screening modalities. However, further risk stratification in diabetic patients is warranted to support a targeted screening strategy with economic viability. Diabetes confers ≈2-fold PDAC risk overall, with the highest relative risk in the first 2–3 years after diagnosis. Strategies using clinical signs (age ≥50–60 years, unintentional weight loss, rapid HbA1c escalation/insulin initiation) and predictive risk scores (e.g., ENDPAC) can triage NOD patients for magnetic resonance imaging/computed tomography (MRI/CT) and endoscopic ultrasound (EUS). A targeted screening approach may allow early diagnosis that could improve the prognosis of PDAC patients. This narrative review aims to synthesize current evidence linking T2DM and PDAC; delineate risk factors within diabetes populations; appraise predictive models and biomarkers for differentiating PCRD from typical T2DM; outline pragmatic, risk-adapted screening strategies, especially for NOD, and identify additional areas where further research is needed.

## 1. Introduction

Pancreatic ductal adenocarcinoma (PDAC) comprises more than 90% of pancreatic malignancies and continues to demonstrate a poor prognosis, with a 5-year survival rate reaching 15% despite advances in surgical and systemic therapy [1,2,3,4]. The lethality of PDAC stems from several factors: rapid progression, lack of specific symptoms in early stages, an anatomic location that complicates early detection, and limited efficacy of systemic therapies [5,6]. In this context, there is intense interest in strategies that shift diagnosis to an earlier, potentially curable stage. However, universal screening is not feasible or cost-effective because PDAC is relatively rare in the general population, and available tests are imperfect. Consequently, the search has therefore turned toward high-risk groups in whom pretest probability is elevated [7,8,9,10].

Type 2 diabetes mellitus (T2DM) represents a suitable population for risk-adapted early detection for two main reasons. First, long-standing T2DM is associated with a modestly increased risk of PDAC, likely due to metabolic and inflammatory mechanisms that promote carcinogenesis [11,12]. Second, new-onset diabetes (NOD) in older adults may indicate a paraneoplastic phenomenon driven by subclinical PDAC, referred to as pancreatic cancer–related diabetes (PCRD), which is classified as type 3c diabetes [2,12]. Several studies showed that the relative hazard of PDAC is highest within the first 2–3 years following diabetes diagnosis, when a subset of tumors could still potentially be resectable [13,14,15,16].

This complex bidirectional relationship complicates epidemiologic inference, but it also highlights a possible clinically actionable window. In clinical practice, the appearance of rapidly worsening glycemic control, unexpected weight loss, or the need to initiate insulin soon after diabetes diagnosis in individuals aged ≥50–60 years should raise suspicion for occult PDAC and consider further evaluation [14,15,17,18]. Recent studies have translated this concept into risk-stratification tools such as the ENDPAC score and large-scale prediction models using primary-care electronic records (QResearch), as well as integrated clinical-genetic models in prospective cohorts [19,20,21,22,23].

An optimal management framework for the assessment and prioritization of diabetic patients related to pancreatic cancer risk remains a challenge. Developing standardized protocols that integrate clinical, biochemical, and imaging parameters is needed to ensure the timely identification of high-risk individuals and to facilitate early detection of PDCA. There are still pending questions, particularly regarding the most effective risk stratification criteria, appropriate screening modality, and the balance between early detection benefits and potential harms associated with over-investigation. This review aims to synthesize current evidence across pathophysiology, risk factors, predictive models, biomarkers, and screening modalities, and to outline a risk-adapted, implementable pathway for use in diabetes and primary-care settings. Where possible, we emphasize data derived from large cohorts, guideline statements, and prospective initiatives such as the Early Detection Initiative (EDI), NODES, and DEFEND PRIME [18,19,24,25,26,27].

In contrast to previous reviews, the present article places particular emphasis on NOD as a clinically actionable risk window, integrates evidence from clinical risk models and emerging biomarkers, and focuses on their pragmatic implementation in real-world diabetes and primary care settings.

## 2. Methodology and Study Selection

To ensure an up-to-date synthesis, we performed a comprehensive literature search. Three electronic databases—PubMed, Web of Science, and Scopus—were searched through November 2025. We included peer-reviewed articles published in English, focusing on literature from the past 15 years (January 2010); three earlier studies were also included for their relevance to pathophysiological mechanisms and biomarker research linking T2DM and PDAC. The search strategy used keywords relevant to the manuscript’s focus. These included new-onset diabetes AND pancreatic cancer; risk factors for pancreatic cancer in diabetes; pancreatic ductal adenocarcinoma screening AND type 2 diabetes mellitus; pancreatic ductal adenocarcinoma AND early detection AND biomarkers AND diabetes; pancreatic cancer AND new-onset diabetes AND predictive risk models.

The search included original research articles, narrative and systematic reviews, clinical trials, consensus statements, and relevant clinical guidelines. Additional sources were identified through a manual review of reference lists from key articles. We synthesized and grouped key findings into four major concepts: pathophysiological mechanisms, risk factors in diabetic subgroups, biomarker development, and screening strategies. Therefore, we did not perform a formal meta-analysis or risk-of-bias assessment. The thematic synthesis provided an integrated overview of pathophysiology, risk stratification, biomarkers, predictive models, and potential screening strategies for PDAC in diabetic populations.

## 3. Pathophysiological Link Between T2DM and PDAC

The relationship between T2DM and PDAC is bidirectional [28,29,30,31].

(a) T2DM as a risk factor for PDAC. Long-standing T2DM increases carcinogenic signaling through chronic hyperinsulinemia and the insulin-like growth factor-1 (IGF-1) pathway. Hyperglycemia also leads to oxidative stress and DNA damage, thereby facilitating the clonal expansion of KRAS-mutant cells. These changes and chronic inflammation induce activation of the PI3K/AKT/mTOR signaling pathway, contributing to PDAC development [32]. In addition, adiposity-related inflammation, characterized by elevated pro-inflammatory cytokines (IL-6, TNF-α, leptin, TGF-β), contributes to a favorable microenvironment for carcinogenesis [12,31,33].
(b) PDAC inducing diabetes (PCRD). Subclinical PDAC can induce glycemia impairment months before the tumoral mass is detected by imaging modalities. Tumor-secreted mediators, such as adrenomedullin, and tumor-derived exosomes enriched in proteins and microRNAs can impair β-cell function and lead to peripheral insulin resistance [32,34]. These systemic effects manifest clinically as the characteristic phenotype of weight loss accompanied by rising fasting glucose and HbA1c, a clinical picture not frequently seen in T2DM patients. Recent evidence indicates that tumor-derived exosomes—nano-sized extracellular vesicles—carry factors that interfere with normal glucose metabolism. These disruptions may cause β-cell dysfunction and peripheral insulin resistance, which could explain the early metabolic disturbances in these patients and have an important role in tumor progression and metastasis [34,35]. The exosome hypothesis is particularly compelling because it offers both a mechanism for PCRD and a potential source of circulating biomarkers for early detection [28,36,37].

**Figure 1:**
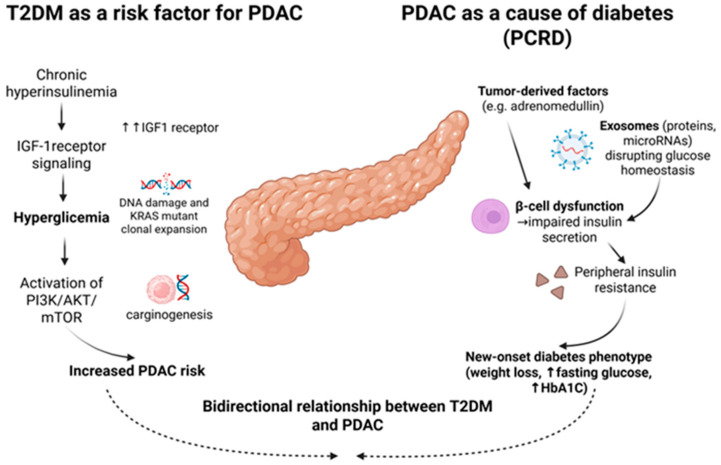
Schematic illustration of the pathophysiological link between type-2 diabetes mellitus and pancreatic ductal adenocarcinoma. Created in BioRender. Bende, F. (2025), https://BioRender.com/ntz72yw (accessed on 14 December 2025).

T2DM, along with obesity and insulin resistance, increases the risk of cancer development. PDAC induces metabolic changes that manifest as diabetes. This overlap creates a clinical challenge and opportunity: determining when diabetes serves as a risk factor for pancreatic cancer versus when it functions as an early sign of PDAC. The bidirectional relationship between T2DM and PDAC is schematically represented in Figure 1.

## 4. Risk Factors for PDAC in Patients with T2DM

The well-established risk factors for PDAC in the general population are: *(i) non-modifiable risk factors*: family history, genetic susceptibility, age; and *(ii) modifiable risk factors*: diabetes mellitus, diet, smoking, alcohol, acute and chronic pancreatitis, and intestinal microbiota [1,8,9,38].

The risk of developing pancreatic cancer among diabetic patients is not uniform. Both the duration and timing of diabetes modify PDAC risk. Individuals with long-standing T2DM (more than 5–10 years) experience a 1.5- to 3-fold increased risk of PDAC compared to non-diabetics [26,39,40,41]. This elevated risk remains after adjustment for confounding factors such as obesity and smoking. The highest relative risk is noted in NOD among older adults, with this subgroup exhibiting a 6–8-fold higher likelihood of PDAC diagnosis within 1–3 years [16,42]. Consequently, NOD in older individuals without a clear precipitant is recognized as an alarm sign for PDAC, prompting efforts to identify which patients might benefit from screening.

In addition to diabetes duration, metabolic and clinical features of the diabetic patient further impact cancer risk. Modifiable lifestyle and comorbid risk factors, including cigarette smoking, heavy alcohol consumption, longstanding obesity, and chronic pancreatitis, can increase the baseline PDAC risk [18,38]. Family history and genetic predisposition also play significant roles in diabetes: those with germline mutations, such as in BRCA2 or ATM, or with multiple first-degree relatives with PDAC, comprise a very high-risk group. Such individuals are often considered for specialized screening programs [8,9,43].

The influence of anti-diabetic medications on pancreatic cancer development remains controversial. Although some evidence suggests that insulin may elevate cancer risk and metformin may reduce it, current findings have not reached a clear consensus [13,44,45].

**Figure 2:**
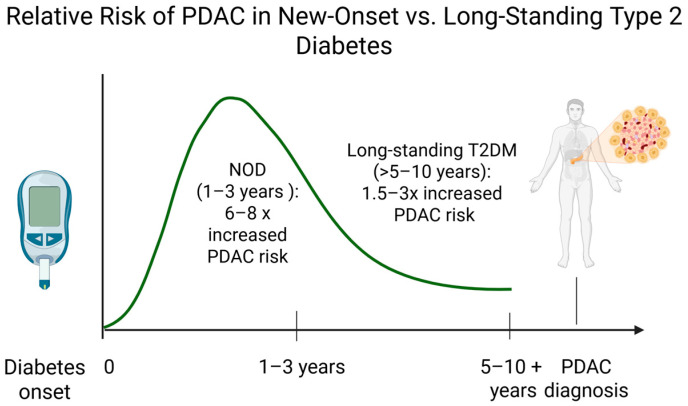
Temporal relationship between diabetes duration and relative risk of pancreatic ductal adenocarcinoma. Created in BioRender. Bende, F. (2025). https://BioRender.com/6wkjwah (accessed on 18 December 2025).

Patients with T2DM, whether newly diagnosed or of long duration, have an elevated risk of PDAC. NOD in an older adult, especially accompanied by unexplained weight loss or deteriorating glycemic control, is an alarm sign with a higher short-term risk for PDAC. Large cohort studies [14,15,20,46] support this dual nature of the diabetes–pancreatic cancer relationship: predisposing factor versus early symptom of PDAC. Clinical vigilance for these risk factors in the T2DM population could lead to earlier diagnosis and improved outcomes. Figure 2 shows a schematic illustration of the relative risk of developing PDAC in the diabetic population, stratified by diabetes duration.

## 5. Biomarkers for Early PDAC Detection in Patients with T2DM

Biomarkers include metabolic markers, tumor antigens, cytokines, and genetic/molecular markers. Studies have demonstrated the potential of several biomarkers for early PDAC detection, representing a further step toward better risk stratification among diabetic patients [47,48,49,50].

Among these biomarkers, CA19-9 remains the most widely used. However, its sensitivity in early PDAC is limited, and false positives occur with cholestasis and poorly controlled diabetes. One study proposed a CA19-9 cut-off of 98.4 U/mL in diabetic patients with a mean HbA1c of 10.0%, achieving improved specificity (70% sensitivity and 96.5% specificity) compared with the conventional 37 U/mL cut-off, but prospective validation in NOD cohorts is needed [51].

Protein biomarker panels hold promise. Notably, the combination of adiponectin and interleukin-1 receptor antagonist (IL-1Ra) achieved an AUC of 0.91 (CI: 0.84–0.99) in distinguishing PCRD from individuals with NOD [48].

Metabolomics has yielded reproducible signatures of amino acids and lipid species that, when combined with CA19-9, achieve AUCs of 0.90–0.95 for differentiating PDAC from chronic pancreatitis and T2DM/NOD. Several studies report encouraging performance for stage-I disease [52]. Exosome-based biomarkers are an important field of research due to their specificity, as they reflect the characteristics of their parent cells and have the potential to distinguish PCRD from T2DM [36].

Additional biomarkers considered valuable for diagnosing PDAC in diabetic patients are listed in Table 1 [18,47]. None of these biomarkers has been prospectively validated as a reliable marker for early pancreatic cancer detection.

While several circulating and molecular indicators show promise for early pancreatic cancer diagnosis, significant limitations prevent their frequent clinical use.

Circulating tumor DNA (cfDNA), exosomal RNA and protein signatures, metabolomic panels, and multi-analyte assays offer better discrimination but are mostly used in research or specialized centers. These strategies have high analytical costs, require advanced laboratory facilities, and lack clinical decision-making thresholds. Actual cost-effectiveness and false-positive rates in low-prevalence populations are few.

Thus, while pairing biomarker-based risk stratification with clinical models and imaging may enhance performance, this approach is not yet suited for routine diabetes screening. Deployment will depend on prospective validation, test standardization, and clinical utility in risk-enriched populations.

## 6. Predictive Models for PDAC Risk in Adults with T2DM

By combining clinical features, risk factors, and lab tests/biomarkers, several predictive risk models have been developed to stratify PDAC risk among adults with T2DM, particularly NOD, as presented in Table 2.

One of the earliest models was developed by Boursi et al. [62], derived from a large U.K. primary care database (THIN database). The model included clinical parameters (age, BMI, and recent weight loss, smoking status, duration of diabetes, use of proton pump inhibitors, metformin, etc.) and laboratory results (hemoglobin, creatinine, alkaline phosphatase, cholesterol, HbA1c). The final risk score had an area under the ROC curve (AUC) of 0.82 for distinguishing those who would be diagnosed with PDAC. At a 3-year risk threshold of 1%, it had a 44.7% sensitivity and 94% specificity (PPV ~2.6%) for PDAC.

Sharma et al. [64] proposed the ENDPAC model (Enriching New-Onset Diabetes for Pancreatic Cancer), focusing on three variables: (1) weight change after diabetes onset, (2) change in blood glucose (A1c) after onset, and (3) months between diabetes onset and diagnosis. The model had AUC of 0.87 with 80% sensitivity and specificity for patients scoring ≥3 points.

Clift et al. developed three models (Cox proportional hazards modelling; XGBoost; artificial neural networks) using data from 253,766 patients with NOD in England (QResearch primary care database) [20]. It included demographics (age, sex, BMI), select comorbidities (e.g., prior venous thrombosis), medications (e.g., digoxin), routine lab tests (HbA1c, ALT, creatinine, hemoglobin, platelets), and recent symptoms (weight loss, abdominal pain, jaundice, dyspepsia). The Cox model showed good discrimination, with a Harrell’s C-index of 0.802 (95% CI: 0.797–0.817), and was internally and externally validated.

More recently, Claridge et al. [27] initiated a feasibility study, DEFEND PRIME, to implement risk scoring in NOD patients aged ≥50 years in UK primary care. It focuses on calculating ENDPAC from electronic records in 20 practices (no performance outcome yet; results pending).

From a clinical perspective, risk stratification models may assist a progressive, risk-adapted approach to pancreatic cancer surveillance in patients with T2DM. In practice, these techniques are not meant for population-wide screening but rather for identifying subgroups who may benefit from additional diagnostic assessment.

ENDPAC is particularly suited for NOD in individuals aged ≥50 years, where scores ≥3 have been associated with a substantially increased short-term risk of PDAC and may reasonably prompt early cross-sectional imaging (contrast-enhanced CT or MRI), followed by EUS in cases with inconclusive findings or persistent clinical suspicion. Its main advantages are its simplicity, utilization of commonly accessible clinical data, and short-term risk assessment. However, ENDPAC is limited to NOD populations and does not account for long-term metabolic changes or broader comorbidity profiles.

In contrast, QResearch-based models provide broader population-level risk estimation, incorporating demographic factors, lifestyle variables, comorbidities, and medication exposure. These tools may be more suitable for long-standing T2DM, enabling longitudinal risk assessment and identification of individuals at moderate cumulative risk. However, their complexity and reliance on large electronic health record datasets may limit immediate bedside applicability, and they are less specific for short-term PDAC prediction.

A pragmatic clinical framework could therefore incorporate initial risk enrichment using simple clinical criteria (age, diabetes duration, unexplained weight loss, glycemic worsening), followed by model-based stratification. High-risk individuals (e.g., ENDPAC ≥3 or top risk percentiles in QResearch-based tools) may be considered for targeted imaging, whereas low-risk patients could continue standard diabetes follow-up. Importantly, such strategies require prospective validation before widespread implementation.

In addition to these risk models, machine-learning models trained on routine biochemical trajectories (HbA1c, triglycerides, creatinine) and age achieved AUCs in the 0.75–0.80 range, suggesting that dynamic signals available in electronic records can meaningfully enrich risk [22]. Sex-specific models (e.g., women with NOD) have also been developed with AUC 0.73 (95% CI 0.68–0.78), reflecting possible differences in baseline risk and treatment patterns [23]. Finally, event-based models triggered at the moment of diabetes progression (e.g., insulin initiation, step-up to combination oral therapy, or sharp HbA1c rise) within integrated health systems stratify 12–36-month PDAC risk with c-index range of 0.70–0.78, supporting a dynamic, event-based screening strategy [65].

## 7. Screening Modalities for PDAC in Diabetic Populations

Multiple guidelines offer evidence-based frameworks for identifying individuals at high risk for early pancreatic cancer and its high-grade precursors, as well as for implementing structured surveillance protocols [8,9,66]. Surveillance for PDAC should be conducted by multidisciplinary teams at centers with relevant expertise [66]. According to these guidelines, candidates for PDAC surveillance include individuals with germline pathogenic variants associated with inherited cancer syndromes and those with a family history indicative of familial pancreatic cancer. The study of Dal Buono et al. underlines the clinical utility of broad multigene panel testing in patients with suggestive personal or family history. Clinicians should systematically implement genetic testing in PDAC care to enhance risk stratification, refine surveillance strategies, and provide access to personalized therapies [67].

The modalities of choice are high-resolution imaging: magnetic resonance imaging with MR cholangiopancreatography (MRI/MRCP) and endoscopic ultrasound (EUS). In addition, fine-needle aspiration or biopsy can be performed during EUS for suspicious lesions. Computed tomography (CT—pancreas protocol) can be used for staging and surgical assessment, but is not recommended for routine screening due to its limited sensitivity for small lesions and radiation exposure.

NOD in a known high-risk individual is considered a significant concern. The American Gastroenterological Association (AGA) expert consensus recommends that the development of diabetes during surveillance should prompt additional diagnostic studies or a reduced interval to the next imaging evaluation, as diabetes may indicate tumor development in this context [8]. Routine screening of unselected individuals with T2DM is not recommended.

Diagnosing PDAC remains challenging, with the lowest early detection rate among major cancers. Substantial evidence demonstrates a correlation between diabetes and PDAC [28,31]. The temporal association between diabetes and PDAC development offers a potential diagnostic window for early detection [47].

Ongoing prospective trials are assessing the feasibility and diagnostic yield of screening in enriched NOD cohorts listed in Table 3. Although definitive outcome data are pending, analogous experience from the CAPS program in genetically high-risk individuals shows that screen-detected lesions are more often resectable and confer markedly improved survival, supporting the principle that earlier detection can change outcomes when appropriate cohorts are identified [27,43,66]. Recent studies explore AI-based screening models utilizing deep-learning algorithms on real-time CT imaging data to identify lesions at an early or premalignant stage [47,68].

**Figure 3:**
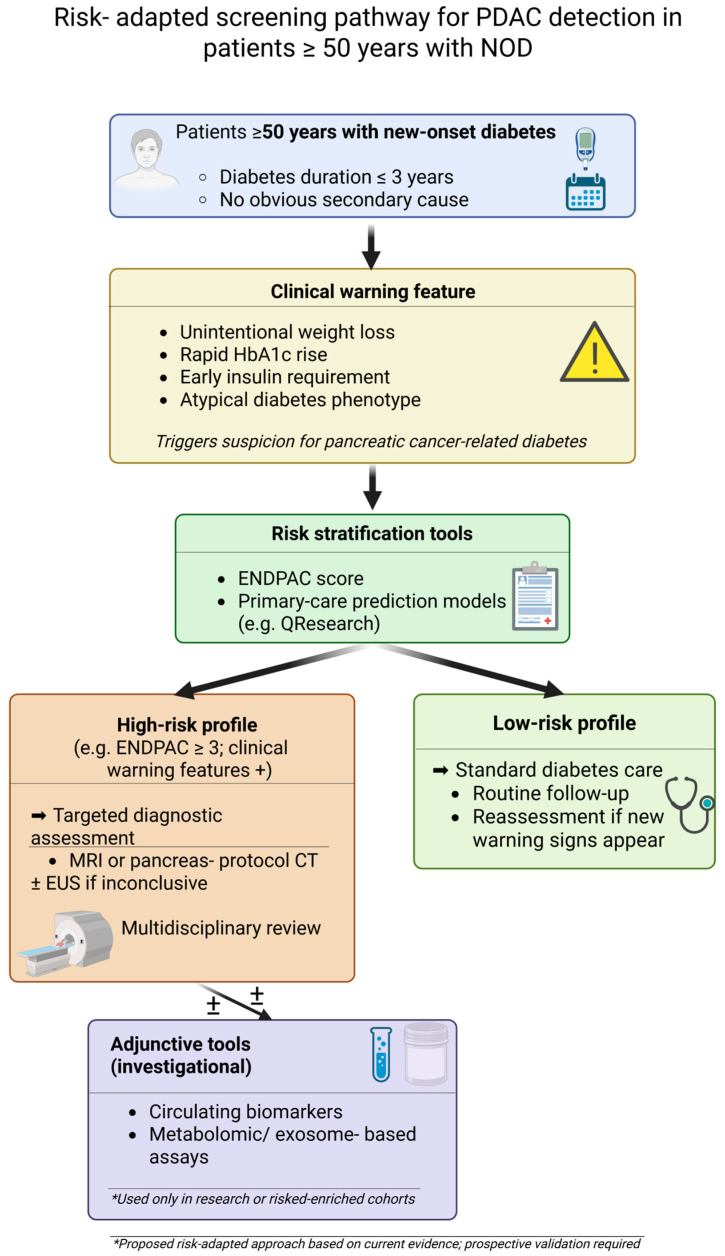
Risk-adapted screening pathway for PDAC detection in patients ≥50 years with NOD. The flowchart illustrates a pragmatic, stepwise approach based on initial clinical risk enrichment, model-based risk stratification (e.g., ENDPAC), and targeted imaging (MRI/CT and EUS) for high-risk individuals, while low-risk patients continue standard diabetes care. The pathway reflects current evidence and requires prospective validation. Created in BioRender. Ghiuchici, A. (2025). https://BioRender.com/35pqfug (accessed on 25 December 2025).

A simplified overview of this risk-adapted screening and triage pathway for patients aged ≥50 years with NOD is shown in Figure 3.

## 8. Future Perspectives

The connection between new-onset diabetes and pancreatic cancer has opened an important window for earlier detection, and most future progress will likely come from better risk stratification. Current clinical tools such as ENDPAC are helpful, but they remain imperfect, and ongoing prospective cohorts—including the NODES study—are expected to refine these models using real-world longitudinal data [24]. These studies follow large numbers of adults with new-onset diabetes for several years and will clarify which combinations of age, weight change, glucose dynamics and laboratory parameters are most predictive of an underlying pancreatic malignancy.

A second direction involves the development of multi-analyte blood tests. Traditional markers like CA19-9 have limited sensitivity in asymptomatic disease, but newer components—such as thrombospondin-1 (which was shown to decrease up to two years before cancer diagnosis, especially in patients with diabetes [69])—could complement existing scores. In parallel, research on tumor-derived exosomes and circulating diabetogenic factors suggests that specific proteins or RNA transcripts released by pancreatic tumors may help distinguish PCRD from ordinary T2DM [31]. If these markers continue to show reproducible results, they may become part of a “second-step” test applied only to patients already flagged as high-risk.

Another important area is the integration of imaging in targeted surveillance programs. Early MRI or EUS has already been shown to detect asymptomatic lesions in familiar high-risk groups, and the same model may eventually be adapted for selected patients with NOD. Cost-effectiveness analyses suggest that surveillance becomes reasonable once a subgroup reaches an estimated risk of around 1% over three years [70]. If future models can reliably identify such patients, periodic MRI and EUS could become the standard of care in this newly recognized high-risk population. Simplified pragmatic screening approaches based only on age and diabetes duration could offer practical advantages in real-world clinical settings. For instance, individuals aged ≥ 60 years with type 2 diabetes diagnosed within the preceding 2–3 years could be considered eligible for PDAC screening and undergo first-line pancreatic imaging evaluation (e.g., MRI), having in mind that the highest relative risk is noted in NOD patients. While such an approach may facilitate early detection, it currently lacks prospective validation, raises economic concerns, and therefore cannot yet be recommended for widespread implementation.

Artificial intelligence is also expected to play a growing role. Machine-learning models trained on electronic health records have already matched the performance of classical clinical scores in predicting pancreatic cancer among individuals with diabetes [22,62]. As larger datasets accumulate, AI may identify subtle temporal trends—such as patterns of weight loss or glycemic instability—that clinicians would not detect manually.

A fourth perspective lies in metabolic prevention. Several mechanistic studies show that hyperglycemia, hyperinsulinemia and chronic inflammation promote pancreatic carcinogenesis, while tumor-secreted molecules worsen glucose metabolism [31]. Improving insulin sensitivity, whether through lifestyle intervention, weight loss or medications such as metformin, might theoretically reduce cancer risk, although prospective trials are still needed. Understanding this metabolic–oncologic interface may also yield new therapeutic targets for preventing or delaying PDAC development in high-risk individuals.

Finally, the success of any early-detection strategy will depend on multidisciplinary coordination. The AGA clinical update already emphasizes that pancreatic screening should be concentrated in experienced centers, and that early investigation is justified when NOD occurs in individuals with genetic predisposition or pancreatic risk factors [8]. As evidence grows, similar structured pathways may be extended to specific metabolic subgroups, especially adults over 50 with new diabetes and unexplained weight loss. Educating primary-care clinicians and diabetologists about this association will be essential for timely referral.

Overall, future progress will come from combining risk models, blood-based biomarkers, advanced imaging and real-time clinical data. If these elements are integrated effectively, they may finally allow pancreatic cancer to be diagnosed during the narrow window in which it is still curable.

## 9. Conclusions

The link between T2DM and PDAC is complex, but one key message stands out: while long-standing diabetes slightly increases cancer risk, NOD after the age of 50 (especially when accompanied by weight loss or rapidly worsening glycemic control) can sometimes be an early sign of an underlying tumor. Most people with T2DM will never develop PDAC, so universal screening is neither practical nor recommended. Instead, attention should focus on those with late-onset or atypical diabetes patterns, particularly when additional risk factors such as smoking, chronic pancreatitis or a family history of pancreatic cancer are present.

Emerging risk scores and biomarkers are promising tools for selecting the patients who may benefit from targeted imaging, but they still require further validation. Until then, good communication between primary care, diabetologists and gastroenterologists and a high index of suspicion in the right clinical context remain essential. At the population level, prevention and optimal management of obesity and T2DM may also contribute to lowering the future burden of pancreatic cancer.

Future research should focus on prospectively validating risk stratification models in well-defined diabetic subpopulations, especially those with NOD and high-risk traits. Studies that combine clinical characteristics, circulating biomarkers, and modern imaging modalities into multimodal prediction frameworks are essential. Health-economic evaluations setting cost-effective targeted imaging and surveillance thresholds are crucial. Finally, standardized, risk-adapted therapeutic pathways matched with real-world diabetes care processes are needed to translate growing data into meaningful pancreatic cancer–related mortality reductions.

## Figures and Tables

**Table 1:** Biomarkers for PDAC in patients with diabetes mellitus.

Biomarker	Specificity	Sensitivity	Author/Year
Islet amyloid polypeptide (IAP)	92%	39%	Chari et al. 2001 [53]
Soluble receptor 2 of tumor necrosis factor-(sTNF-R2)	N/A (no diagnostic cut-off)	N/A (no diagnostic cut-off)	Grote et al. 2012 [54]
Osteoprotegerin (OPG)	73.9%	68%	Shi et al. 2014 [55]
Vanin-1 (VNN1)	N/A (candidate gene)	N/A (candidate gene)	Kang et al. 2016 [56]
Angiopoietin-like protein 2 (ANGPTL2)	AUC 0.906 (*p* < 0.001, 95% CI: 0.815–0.997; *p* < 0.001)	AUC 0.906 (*p* < 0.001, 95% CI: 0.815–0.997; *p* < 0.001)	Yoshinaga et al. 2018 [57]
S100 calcium-binding protein A8 (S100 A8)	N/A 1.10 (95% CI, 1.04–1.16; *p* = 0.001)	N/A 1.10 (95% CI, 1.04–1.16; *p* = 0.001)	Liao et al. 2023 [58]
Matrix metalloproteinase 9 (MMP9)	N/A (no added value over CA19-9)	N/A (no added value over CA19-9)	Moz et al. 2016 [59]
Circulating RNA	N/A (six-miRNA panel AUC = 0.887)	N/A (six-miRNA panel AUC = 0.887)	Dai et al. 2016 [60]
Plasma free amino acid profile	92.7%	66.7%	Roberts et al. 2014 [61]

N/A, not available; AUC, area under the curve; CA19-9, Carbohydrate Antigen 19-9; miRNA, circulating microRNAs.

**Table 2:** Predictive risk models in NOD.

Author/Year	Study Type; Sample Size (*n* = NOD)	Key Predictors	Performance
Boursi et al. 2017 [62]	retrospective cohort study; *n* = 109,385	Age, BMI, change in BMI, smoking, use of PPI, antidiabetic medications, HbA1c, cholesterol, Hb, Cre, ALP	AUC 0.82
Dong et al. [63]	matched case–control study *n* = 171	BMI, age of DM onset, HBV infection, T. Bil, ALT, Cre, APO-A1, WBC	AUC 0.82
Sharma et al. 2018 [64]	retrospective cohort study; *n* = 1561	Age, weight loss, glycemia	AUC 0.87
Clift et al. 2024 [20]	retrospective cohort *n* = 253,766	Age, sex, BMI, comorbidities, medications, HbA1c, ALT, creatinine, Hb, PLT; symptoms: abdominal pain, weight loss, jaundice, heartburn, indigestion or nausea	Harrell’s C-index 0.802

AUC, area under the curve; NOD, new-onset diabetes; BMI, body mass index; DM, diabetes mellitus; HBV, hepatitis B virus; T. Bil, total bilirubin; ALT, alanine aminotransferase; Cre, creatinine; APO-A1, apolipoprotein-A1; WBC, white blood cell; PPI, proton pump inhibitors; HbA1c, hemoglobin A1c; Hb, hemoglobin; ALP, alkaline phosphatase.

**Table 3:** Clinical trials exploring screening methods in diabetic patients.

Study	ClinicalTrials.Gov ID Identifier
EDI (Early Detection Initiative for Pancreatic Cancer)	https://clinicaltrials.gov/study/NCT04662879 (accessed on 22 November 2025)
NODES (New Onset of DiabetEs in aSsociation With Pancreatic Cancer)	https://clinicaltrials.gov/study/NCT04164602 (accessed on 22 November 2025)
PANDOME (A PAncreatic Cancer Screening Study in Individuals With New-Onset or DeteriOrating Diabetes Mellitus)	https://clinicaltrials.gov/study/NCT03937453 (accessed on 23 November 2025)

## Data Availability

No new data were created or analyzed in this study. Data sharing is not applicable to this article.

## Abbreviations

The following abbreviations are used in this manuscript:

AKT	Protein kinase B (PKB)
CA19-9	Carbohydrate Antigen 19-9
CT	Computed tomography
DNA	Deoxyribonucleic Acid
ENDPAC	Enriching New-Onset Diabetes for Pancreatic Cancer
EUS	Endoscopic ultrasound
HbA1c	Hemoglobin A1C
IGF-1	Insulin-like Growth Factor 1
IL-6	Interleukin-6
KRAS	KRAS proto-oncogene
MRI	Magnetic resonance imaging
MRCP	Magnetic resonance cholangiopancreatography
mTOR	Mammalian Target of Rapamycin
NOD	New-onset diabetes
PCRD	Pancreatic cancer–related diabetes
PDAC	Pancreatic ductal adenocarcinoma
PI3K	Phosphoinositide 3-kinase
T2DM	Type 2 diabetes mellitus
TNF-α	Tumor Necrosis Factor alpha
